# Association between white matter hyperintensity load and grey matter atrophy in mild cognitive impairment is not unidirectional

**DOI:** 10.18632/aging.202977

**Published:** 2021-04-16

**Authors:** Ashwati Vipin, Benjamin Yi Xin Wong, Dilip Kumar, Audrey Low, Kok Pin Ng, Nagaendran Kandiah

**Affiliations:** 1 National Neuroscience Institute, Singapore, Singapore; 2 Duke-NUS Medical School, Singapore, Singapore; 3 Lee Kong Chian-Nanyang Technological University, Singapore, Singapore

**Keywords:** white matter hyperintensity, mild cognitive impairment, grey matter change, cognition, cerebrovascular disease

## Abstract

Neuroimaging measures of Alzheimer’s disease (AD) include grey matter volume (GMV) alterations in the Default Mode Network (DMN) and Executive Control Network (ECN). Small-vessel cerebrovascular disease, often visualised as white matter hyperintensities (WMH) on MRI, is often seen in AD. However, the relationship between WMH load and GMV needs further examination. We examined the load-dependent influence of WMH on GMV and cognition in 183 subjects. T1-MRI data from 93 Mild Cognitive Impairment (MCI) and 90 cognitively normal subjects were studied and WMH load was categorized into low, medium and high terciles. We examined how differing loads of WMH related to whole-brain voxel-wise and regional DMN and ECN GMV. We further investigated how regional GMV moderated the relationship between WMH and cognition. We found differential load-dependent effects of WMH burden on voxel-wise and regional atrophy in only MCI. At high load, as expected WMH negatively related to both ECN and DMN GMV, however at low load, WMH positively related to ECN GMV. Additionally, negative associations between WMH and memory and executive function were moderated by regional GMV. Our results demonstrate non-unidirectional relationships between WMH load, GMV and cognition in MCI.

## INTRODUCTION

Mild cognitive impairment (MCI) is considered a high-risk prodromal stage of Alzheimer’s disease (AD) with more than a third of patients at increased risk of progression to dementia [1]. A diagnosis of MCI is characterized by objective cognitive impairment, related to memory as well as other cognitive domains, but without functional impairment that interferes with activities of daily life [2, 3]. Additionally, the presence of cerebrovascular risk factors are also thought to place cognitively normal individuals at increased risk of progression along the AD spectrum [4].

Indeed, studies show that small-vessel cerebrovascular disease, represented by surrogate Magnetic Resonance Imaging (MRI) measures particularly white matter hyperintensities (WMH) [5, 6], is an important risk factor for the clinical manifestation of MCI and progression to dementia [7–9]. Moreover, high WMH volume itself has been associated with greater risk of progression from cognitively normal to MCI as well as medial temporal lobe atrophy and cognitive impairment in AD [2, 10, 11]. Specifically, WMH-related global reductions in grey matter volume (GMV) together with frontal and parietal-lobe specific structural alterations have been widely observed in AD [12]. Previous studies have demonstrated that WMH plays a role in grey matter (GM) structural changes and cognitive decline even at the MCI stage [13–18]. Structural decline has been shown to involve temporal and frontal regions comprising the AD-related default mode and executive control networks [19–21]. Recent findings have shown that even at low levels of WMH in cognitively unimpaired middle-aged individuals, higher WMH lesion volume is significantly associated with a widespread pattern of lower GMV in temporal, frontal, and cerebellar areas [22]. However, results have been mixed with some studies suggesting that higher WMH is associated with higher network-based GMV and functional connectivity involving the default mode and executive control networks in the cognitively normal, MCI and AD [20, 23, 24]. This relationship thus needs further elucidation in early disease stages. While our previous work has also shown that derogatory influences of WMH on GMV are most widespread at the MCI stage, compared to both cognitively normal and AD stages [25], the possible differential effects of WMH load on GMV in the cognitively normal and MCI remain to be elucidated to allow for development of strategies to reduce irreversible structural and cognitive damage.

Greater baseline WMH burden is also predictive of accelerated neuro-cognitive decline as well as increase in clinical dementia rating longitudinally [26]. Additionally, cross-sectional studies illustrate associations between high WMH and decline in memory and executive function in healthy controls and MCI [13, 27, 28]. Global and regional cortical thickness have also been shown to mediate the relationship between WMH and global cognition in cognitively healthy individuals, MCI and AD patients [11]. Additionally, GMV has also been shown to mediate the association between WMH burden with both executive function and memory, in mixed populations of individuals with cardiovascular risk factors and AD patients [29]. However, few studies have explored how differing loads of WMH can influence relationships between WMH, GMV and cognition. The extent to which these associations are present in the early stages of disease involving both cognitively normal elderly and MCI, especially in the Asian context, has not yet been fully assessed.

In light of these uncertainties, we sought to assess the load-dependent influence of WMH on whole-brain voxel-wise and region of interest-based GMV and cognition in cognitively normal individuals and individuals with MCI from an Asian cohort. Based on prior evidence of the influence of WMH on default mode and executive control networks, we assessed the effect of WMH on major regions comprising these networks. We hypothesized that increasing WMH load would result in lower voxel-wise and regional GMV in the cognitively normal and MCI stages. We also investigated the influence of WMH load on memory and executive function. We hypothesized that increasing load of WMH would be related to greater impairment in domains of memory and executive function. Additionally, we also examined the mediating and moderating effect of GMV on the relationship between WMH and cognition.

## MATERIALS AND METHODS

### Study participants

Cognitively normal individuals and participants with a diagnosis of MCI were recruited from tertiary neurology centres in Singapore between August 2013 and August 2018. Inclusion criteria included diagnosis of MCI based on the NIA-AA criteria [30]. Subjects with MCI were required to have cognitive symptoms, deficits on neuropsychological evaluation, CDR of 0.5 and to not meet criteria for dementia. For cognitively normal subjects, inclusion criteria included absence of subjective cognitive symptoms and a CDR of 0. Exclusion criteria included: 1) a history of alcohol or drug abuse; 2) a current or known history of major depression; 3) comorbid neurodegenerative disease such as Parkinson’s disease; 4) history of stroke; 5) presence of contraindications to MRI.

Participants also underwent APOE genotyping using TaqMan SNP genotyping assays on ABI 7900HT PCR system (Applied Biosystems, Foster City, CA). APOE genotype assignments were performed as described [31].

Informed consent for both studies was sought from each patient according to the Declaration of Helsinki and local clinical research regulations. The study was granted approval by the Singhealth Centralized Review Board. Following quality control, we included 90 cognitively normal individuals and 93 participants with MCI in our study.

### Neuropsychological assessments

Patients underwent a standardized battery of neuropsychological assessments administered by trained research staff. Cognitive information collected examined domains of 1) episodic memory, assessed using Alzheimer’s Disease Assessment Scale (ADAS)–Cognitive 10-word delayed recall [32] and ADAS-Immediate Recall [33]; and 2) executive function, assessed using Frontal Assessment Battery [34] and Color Trails 2 [35]. Measures of global cognition included the Mini-Mental State Examination [36] and the Montreal Cognitive Assessment [37]. Performance on the individual tasks was transformed into z-scores based on normative scores [38–40].

### Image acquisition

MRI scans were performed on a 3T Prisma Fit System (Siemens, Erlangen, Germany). High resolution T1-weighted MPRAGE (MagnetizationPrepared Rapid Gradient Echo: 192 continuous sagittal slices, TR/TE/TI = 2300/2.28/900ms, flip angle = 8◦, FOV = 256×240 mm2, matrix = 256×240, isotropic voxel size = 1.0×1.0×1.0mm3, bandwidth = 200 Hz/pixel and FLAIR (Fluid Attenuated Inversion Recovery) sequences (192 continuous sagittal slices, TR/TE/TI = 5000/387.0/1800ms, flip angle = 15◦, FOV = 256×256 mm2, matrix = 256×256, isotropic voxel size = 1.0×1.0×1.0mm3) were obtained. Scan images were reviewed at acquisition and subjects with motion artifacts and gross pathological findings were excluded.

### Image pre-processing

We used the Computational Anatomy Toolbox (http://dbm.neuro.uni-jena.de/cat12/) protocol in Statistical Parametric Mapping (SPM12) (http://www.fil.ion.ucl.ac.uk/spm/), to process the T1 images for voxel-based morphometry (VBM) analysis. Specifically, all 3D T1-weighted MRI scans were normalized using an affine transformation followed by non-linear registration, corrected for bias field inhomogeneities. Images were then segmented to derive subject-level GM, white matter (WM), and cerebrospinal fluid (CSF) components [41]. The Diffeomorphic Anatomic Registration Through Exponentiated Lie algebra algorithm was used to normalize the segmented scans into the standard MNI space which provides better precision in spatial normalization to the template [42]. Subsequently, the modulation step performed a non-linear deformation on the normalized segmented images. The modulation step provides a comparison of the absolute amounts of tissue corrected for individual differences in brain size. All obtained segmented, modulated, and normalized GM and WM images were then smoothed using an 8-mm full-width-half-maximum isotropic Gaussian smoothing kernel.

### White matter hyperintensity derivation

We used the Lesion Segmentation Toolbox (LST version 2.0.15), a MATLAB (https://www.mathworks.com/?s_tid=gn_logo) and SPM12-based automated tool for WMH detection, to extract binary WMH lesion belief maps [43, 44]. We employed the automated lesion growth algorithm from LST on T1 anatomical and FLAIR images to quantify WMH as reported previously [23]. This algorithm first co-registers the T2 FLAIR to T1 and subsequently segments T1 images into GM, WM and CSF tissue maps. This information is then combined with the co-registered T2 FLAIR images to estimate the WMH lesion belief maps. By thresholding these maps with a pre-determined initial kappa threshold (κ), an initial binary lesion map is obtained and is subsequently grown along voxels that appear hyperintense on the T2 FLAIR image. To define the optimal threshold, T1 and FLAIR images of 10 randomly chosen subjects with mild to severe WMH load were segmented at κ=0.3, κ=0.2 and κ=0.10. After further visual inspection of segmentation results at these threshold levels, the WMH visual raters determined κ=0.10 as the optimal threshold. The total lesion volume in each subject was then obtained using the extract values of interest option in the LST toolbox. Through data-driven means, the obtained lesion volume was categorized into terciles separately i.e. low (0.00-1.57), medium (1.58-3.16) and high (≥3.17) terciles in the cognitively normal and low (0.00-1.49ml), medium (1.50-4.19ml) and high (≥4.20ml) terciles for MCI. Using published methods, total lesion volume was normalized using total intracranial volume and this ratio was log-transformed for use in the statistical analyses [45]. In the following sections, WMH load will thus refer to this log-transformed ratio of WMH over total intracranial volume. Additionally, the lesion probability maps generated by the algorithm were used for lesion filling to correct for the presence of white matter lesions which may lower the estimated grey matter fraction on the T1-weighted images [43]. These lesion-filled images were used for subsequent analyses.

### Region of interest derivation

We applied a multiple seed-based approach to test the association between GMV and WMH load specifically in regions of interest (ROIs) belonging to the Default mode network (DMN) and executive control network (ECN). We selected six ROIs covering the DMN and ECN based on a prior study [46]. The DMN ROIs included the posterior cingulate cortex (PCC) and precuneus (PCN) and the ECN ROIs included the left and right dorsolateral prefrontal cortex (L and R DLPFC) and the left and right posterior parietal cortex (L and R PPC) in standard space. Average GMV from these network ROIs were derived using the MarsBar package in SPM12 [47].

### Statistical analyses

Group differences on participant characteristics across the WMH terciles were assessed using one-way ANOVA analyses for continuous variables and chi-square tests for categorical variables (Table 1A, 1B).

**Table 1A:** Subject demographics: cognitively normal participants.

	**Tercile 1 (n=30)**	**Tercile 2 (n=29)**	**Tercile 3 (n=31)**	***p value***
**Age at diagnosis (years)**	58.80 (6.44)^b,c^	64.9 (6.61)	65.6 (6.18)	*p*<0.001
**Sex (M/F), n**	17/13	17/12	16/15	*p* = 0.852
**Education (years)**	13.30 (4.41)	13.2 (2.92)	12.6 (3.01)	*p* = 0.661
**MMSE**	28.8 (1.35)	28.4 (1.76)	28.7 (1.47)	*p* = 0.616
**MOCA**	28.2 (1.38)	27.3 (2.31)	27.7 (1.81)	*p* = 0.183
**WMH (cm^3^)**	0.92 (0.36)^c^	2.27 (0.51)^c^	8.03 (5.18)	*p*<0.001
**Total GMV (cm^3^)**	580.52 (40.7)	581.38 (47.2)	559.52 (63.4)	*p* = 0.179
**Average SBP (mmHg)**	126.59 (17.36)	127.24 (14.89)	136.07 (16.59)	*p* = 0.060
**Total lacunes**	0.20 (0.41)	0.62 (0.97)	1.26 (2.53)	*p = 0.038*
**Total microbleeds, n=38**	0.36 (0.67)	0.15 (0.55)	0.64 (0.93)	*p = 0.245*
**APOE4 Carrier, n**	4/30	3/29	5/31	*p = 0.805*
**ADAS Delayed recall z-score**	0.168 (0.98)	0.166 (0.73)	-0.051 (0.99)	*p = 0.566*
**ADAS Immediate recall z-score**	-0.364 (0.88)	-0.099 (0.85)	-0.208 (1.33)	*p = 0.625*
**Color Trails 2 z-score**	0.450 (0.608)	0.437 (0.95)	0.406 (1.78)	*p =* 0.99
**FAB z-score**	0.683 (0.41)	0.589 (0.45)	0.552 (0.59)	*p =* 0.568

Values represent mean (SD) unless otherwise indicated.

Superscript letters indicate whether group mean was significantly different compared with ^b^Tercile 2, ^c^Tercile 3, based on post-hoc comparisons (p < 0.05) following one-way analysis of variance. Chi-square tests were carried out on sex.

Abbreviations: MMSE, mini-mental state examination; MOCA, Montreal Cognitive Assessment; WMH, white matter hyperintensity; GMV, grey matter volume; SBP, systolic blood pressure; ADAS, Alzheimer’s disease assessment scale; FAB, frontal assessment battery.

**Table 1B:** Subject demographics – mild cognitive impairment.

	**Tercile 1 (n=31)**	**Tercile 2 (n=30)**	**Tercile 3 (n=32)**	***p value***
**Age at diagnosis (years)**	56.40 (6.78)^b,c^	57.09 (6.38)^c^	62.28 (6.75)	*p*<0.001
**Sex (M/F), n**	10/21	17/13	20/12	*p* = 0.040
**Education (years)**	12.80 (3.15)	13.63 (3.80)	11.62 (3.84)	*p* = 0.094
**MMSE**	28.16 (1.44)	27.30 (2.08)	27.46 (1.52)	*p* = 0.115
**MOCA**	26.32 (2.65)	24.96 (3.05)	25.62 (2.82)	*p* = 0.183
**WMH (cm^3^)**	0.72 (0.39)^b,c^	2.55 (0.87)^c^	10.75 (8.01)	*p*<0.001
**Total GMV (cm^3^)**	590.85 (58.39)	605.31 (59.90)	575.05 (51.92)	*p* = 0.116
**Average SBP (mmHg)**	126.94 (18.34)	131.09 (15.74)	131.36 (16.32)	*p* = 0.514
**Total lacunes**	0.35 (0.66)	0.4 (0.62)	2.13 (2.43)	*p<0.001*
**Total microbleeds, n=68**	0.16 (0.51)	0.36 (0.64)	1.54 (3.98)	*0.132*
**APOE4 Carrier, n**	5/28	8/26	6/28	*p = 0.513*
**ADAS Delayed recall z-score**	0.311 (1.11)	1.17 (1.85)	0.772 (0.99)	*p* = 0.056
**ADAS Immediate recall z-score**	0.435 (1.29)	0.998 (1.59)	0.848 (1.55)	*p* = 0.315
**Color Trails 2 z-score**	-0.092 (0.97)	0.127 (0.84)	-0.78 (3.49)	*p =* 0.27
**FAB z-score**	0.395 (0.68)	0.371 (0.78)	0.113 (1.10)	*p =* 0.38

Values represent mean (SD) unless otherwise indicated.

Superscript letters indicate whether group mean was significantly different compared with ^b^Tercile 2, ^c^Tercile 3, based on post-hoc comparisons (p < 0.05) following one-way analysis of variance.

Chi-square tests were carried out on sex.

Abbreviations: MMSE, mini-mental state examination; MOCA, Montreal Cognitive Assessment; WMH, white matter hyperintensity; GMV, grey matter volume; SBP, systolic blood pressure; ADAS, Alzheimer’s disease assessment scale; FAB, frontal assessment battery.

### Association between WMH and voxel-wise GMV

To assess the positive and negative effect of WMH on voxel-wise GMV, we built a voxel-wise multiple regression model with GMV as the dependent variable and log-transformed WMH and total intracranial volume ratio as the independent variable of interest. Age at diagnosis and sex were added as covariates to the analysis. The GM clusters showing significant effect of WMH were examined using a threshold of uncorrected p<0.001 and a minimum cluster size of 100 voxels [25, 48]. Significant GM clusters were identified using the Automated Anatomical Labelling atlas. This analysis was conducted separately for MCI and cognitively normal subjects.

### Associations between default mode network and executive control network regions of interest GMV and WMH load

Pearson’s correlation analysis was used to assess the association between ROI GMV and WMH load in each tercile separately. Significant effect of WMH is reported at p<0.05 following False Discovery Rate (FDR) correction for multiple comparisons across the six regions of interest and then at a lower uncorrected threshold of p<0.05. Partial correlation analysis was used to assess the association between ROI GMV and WMH load while controlling for age at diagnosis, sex, hypertension status, systolic blood pressure, history of diabetes and hyperlipidaemia, separately.

### Associations between WMH load and cognition

Pearson’s correlation analysis was used to assess the association between WMH load and cognition separately at each WMH tercile. Cognitive test z-scores comprising the ADAS delayed recall and ADAS immediate recall as well as Color Trails 2 and Frontal assessment battery were used in the analyses to represent memory and executive function assessments, respectively.

### Mediation and moderation effect of WMH load on the association between ROI GMV and cognition

A mediation analysis was conducted to test whether ROI GMV mediated the effect of WMH on cognition. Individual mediation models were fitted for each GMV ROI and each cognitive test at terciles showing significant associations between WMH load and ROI GMV. Specifically, each model included WMH load as the predictor, ROI GMV as the mediator, and cognitive test scores as the outcome. Each model controlled for age at diagnosis and sex. The mediation model was significant if the relationship between WMH load and cognition was reduced when controlling for the mediator. The absence of a significant direct relationship between WMH and cognition after including the mediator was considered a full mediation. On the other hand a significant direct relationship after including the mediator was considered a partial mediation.

A moderation analysis was conducted to assess whether ROI GMV moderated the relationship between WMH and cognition. For this, we carried out a linear regression analysis at each WMH tercile. Cognitive test z-scores comprising the ADAS delayed recall, ADAS immediate recall, Color Trails 2 and Frontal assessment battery were used in the linear regression model to represent memory and executive function assessments. An interaction term between ROI GMV and WMH load was included to assess the moderation effect. Age at diagnosis and sex were added as nuisance covariates in the linear regression model. Multiple comparisons correction across the six ROIs and four cognitive tests was conducted using False Discovery Rate (FDR) correction for multiple comparisons and then at a lower uncorrected threshold of p<0.05.

The statistical analyses for mediation models was carried out using the Statistical Package for Social Sciences (SPSS, Inc; Chicago, IL, USA) version 23.0 macro PROCESS [49]. Effect size estimation was applied using bias-corrected bootstrap estimation with 5,000 resamples [50]. A bias-corrected 95% bootstrapped confidence interval (CI) that did not contain zero indicated a significant effect [50]. All other statistical analyses were conducted using R 3.0.3 (R CoreTeam, 2014) with RStudio (RStudio Team, 2012).

## RESULTS

90 cognitively normal participants (Table 1A) with a mean age of 63.1 (SD 7.04), mean WMH of 3.80 (SD 4.35) and 93 MCI participants (Table 1B) with a mean age of 58.65 (SD 7.09) years and mean WMH volume of 4.76 (SD 6.44) cm^3^ were studied. Participant demographics and cognitive characteristics categorized by tercile of WMH are summarized in Table 1A, 1B. Participant age was significantly different between WMH terciles. Participants did not differ on disease severity as indicated by their comparable global cognition, memory and executive function profiles (Table 1A, 1B).

### Associations between voxel-wise grey matter volume and WMH load in the cognitively normal and mild cognitive impairment

In the cognitively normal, WMH load was not associated with GMV at tercile 1 or tercile 3 (Table 2A). Only within tercile 2, higher WMH load was associated with lower GMV in the left precuneus, right middle temporal gyrus, right superior parietal and bilateral lingual gyrus (p<0.001, minimum cluster size = 100 voxels).

**Table 2:** Associations between white matter hyperintensity load and voxel-wise grey matter volume in normal controls and mild cognitive impairment.

**A) Normal controls**	**Region**	**Peak t-statistics**	**Cluster size**	**MNI coordinates**
**Negative association between WMH load and GMV**				
Tercile 2	Left rectus Left precuneus; right superior parietal gyrus Right middle temporal gyrus Right superior parietal; right precuneus Left lingual gyrus Right lingual gyrus	5.60 5.01 4.69 4.60 4.58 4.44	193 168 341 329 208 146	-9 26 -14 -14 -75 56 63 -48 -4 16 -60 63 14 -69 -10 -20 -60 -12
**B) Mild cognitive impairment**	**Region**	**Peak t-statistics**	**Cluster size**	**MNI coordinates**
**Positive association between WMH load and GMV**				
Tercile 1	Left angular gyrus	4.92	196	-48 -64 28
**Negative association between WMH load and GMV**				
Tercile 1	Right postcentral gyrus Right middle/superior frontal gyrus Right middle cingulum Right anterior cingulum Left middle/inferior frontal gyrus Left superior/medial frontal gyrus Right lingual gyrus Left inferior frontal, triangular part	5.83 5.60 5.49 4.75 4.57 4.50 4.40 4.25	805 277 428 183 221 118 117 190	46 -16 38 33 6 57 15 -39 40 3 39 22 -40 44 -10 -10 42 24 26 -90 -16 -36 27 6
Tercile 3	Left supramarginal gyrus Right superior parietal gyrus; right postcentral gyrus; right inferior parietal gyrus Left inferior parietal gyrus Right middle/inferior frontal gyrus Right precuneus; right middle cingulum Right angular gyrus	6.08 5.08 4.49 4.39 4.13 3.78	909 1172 388 127 207 102	-54 -34 32 26 -57 58 -39 -54 52 42 28 30 9 -46 42 45 -68 36

All voxel-wise analyses controlled for age at diagnosis and sex.

Abbreviations: WMH, white matter hyperintensity; GMV, grey matter volume.

**Figure 1:**
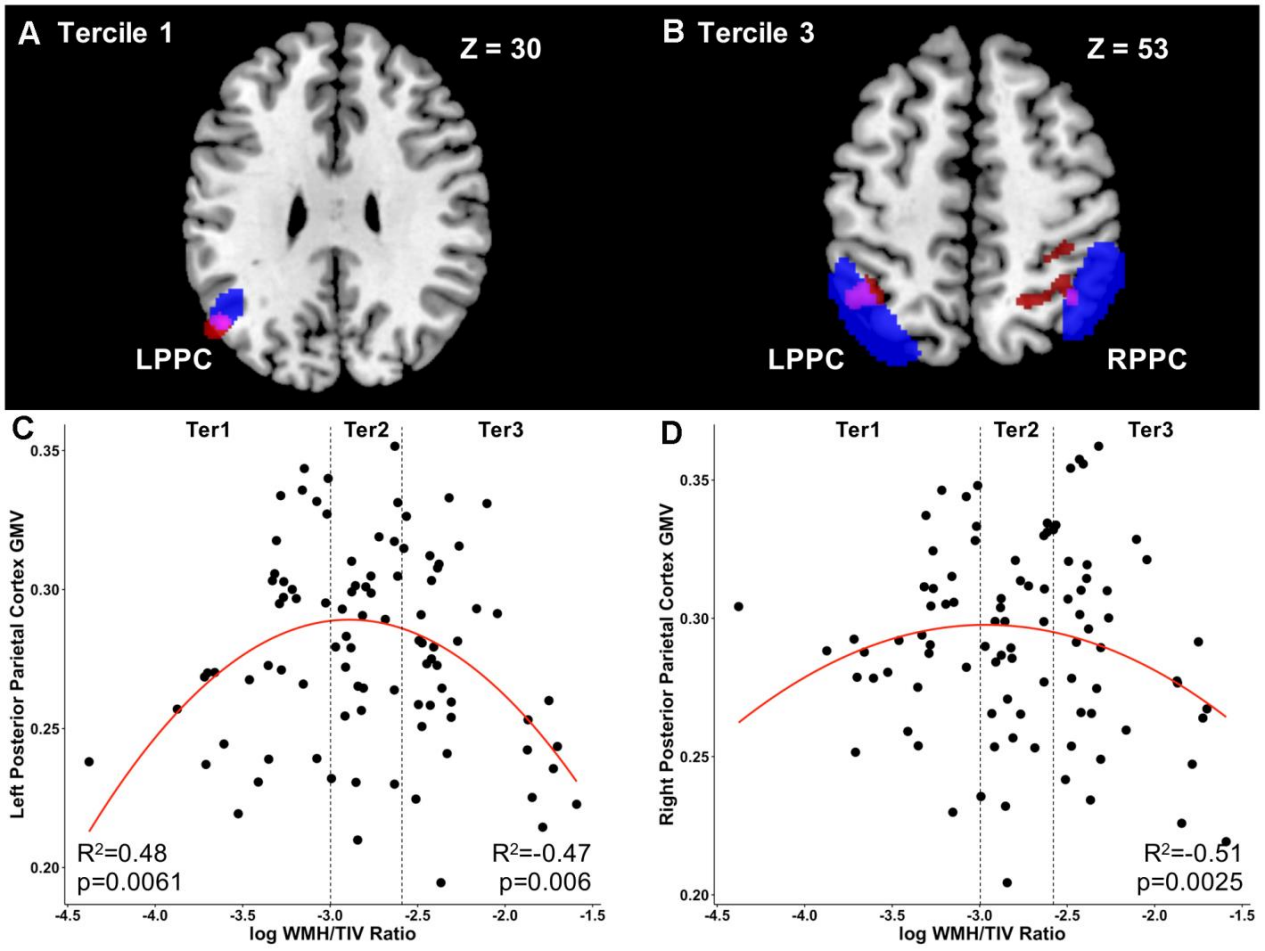
**Load-dependent differential relationships between grey matter and white matter hyperintensity load in mild cognitive impairment.** (**A**) The brain slice shows the positive association between voxel-wise GMV and WMH load at Tercile 1 in the left parietal cortex and angular gyrus (red) overlapping with the left posterior parietal cortex ROI (blue). Overlapping region is shown in purple. (**B**) The brain slice shows the negative association between voxel-wise GMV and WMH load at Tercile 3 in the left and right parietal cortex (red) overlapping with the right posterior parietal cortex ROI (blue). Overlapping regions are shown in purple. Results are shown at the uncorrected p<0.001 height threshold with an extent threshold of 100 voxels. Results are displayed on representative sections of the MNI template brain. In axial slices, the left side of the image corresponds to the left side of the brain. (**C**) In tercile 1, GMV in the left posterior parietal cortex was positively related to WMH load. No relationship was observed at tercile 2. At tercile 3, on the other hand, the inverse was observed, with increasing WMH load relating to lower GMV in the posterior parietal cortex. (**D**) Similarly, in the right posterior parietal cortex, increasing WMH load in the highest tercile 3 was related to lower GMV. Abbreviations: GMV, grey matter volume; WMH, white matter hyperintensity; Ter, Tercile; L, left; R, right; PPC, posterior parietal cortex; ROI, region of interest.

In MCI participants, at tercile 1, WMH was positively associated with GMV in areas involving the left angular gyrus (Table 2B) including voxels within the posterior parietal cortex (p<0.001, minimum cluster size = 100 voxels; Figure 1A). Additionally, WMH load was also negatively associated with GMV primarily in frontoparietal regions involving the bilateral middle and superior frontal gyrus, bilateral anterior cingulum and left inferior frontal gyrus (p<0.001, minimum cluster size = 100 voxels). At tercile 3, WMH was only negatively associated with GMV in frontoparietal regions involving the right superior parietal gyrus, left inferior parietal, right middle and inferior frontal gyrus, left supramarginal gyrus, right precuneus and right angular gyrus (p<0.001, minimum cluster size = 100 voxels; Figure 1B).

Thus, due to the presence of differential associations between WMH and voxel-wise GMV in MCI in frontoparietal regions involving the ECN and DMN, we further investigated the influence of WMH on ROI-based GMV in these networks as well as on the association between GMV and cognition at the MCI stage only.

### Associations between ROI grey matter volume and WMH load in mild cognitive impairment

In individuals with WMH load within tercile 1, WMH volume was positively associated to GMV in the LPPC (r=0.48; p=0.0061, FDR-corrected p<0.05; Figure 1C). These results remained significant after controlling for age at diagnosis, sex, hypertension status, systolic blood pressure, history of diabetes and hyperlipidaemia. No association between WMH volume and GMV was observed at tercile 2.

However, at tercile 3, a negative relationship was observed between WMH and GMV such that increasing WMH load was associated with lower GMV across both the DMN and ECN: PCC (r=-0.44; p=0.011, FDR-corrected p<0.05), PCUN (r=-0.35; uncorrected p<0.05), LDLPFC (r=-0.42; p=0.016, FDR-corrected p<0.05), RDLPFC (r=-0.38; uncorrected p<0.05), LPPC (r=-0.47; p=0.006, FDR-corrected p<0.05; Figure 1A); RPPC (r=-0.515; p=0.0025, FDR-corrected p<0.05; Figure 1D). Importantly, these results remained significant after controlling for age at diagnosis, sex, hypertension status, systolic blood pressure, history of diabetes and hyperlipidaemia.

### ROI grey matter volume moderates the relationship between WMH load and cognition in mild cognitive impairment

A Pearson’s correlation analyses between log WMH/TIV in each tercile and cognitive performance on episodic memory (ADAS delayed recall, ADAS immediate recall) and executive function (color trails 2, frontal assessment battery) was carried out at the MCI stage. No significant associations were observed between low, medium or high WMH and cognitive performance.

We found no mediation effect of DMN or ECN ROI GMV on the relationship between WMH load and cognition for any of the cognitive tests in MCI.

**Figure 2:**
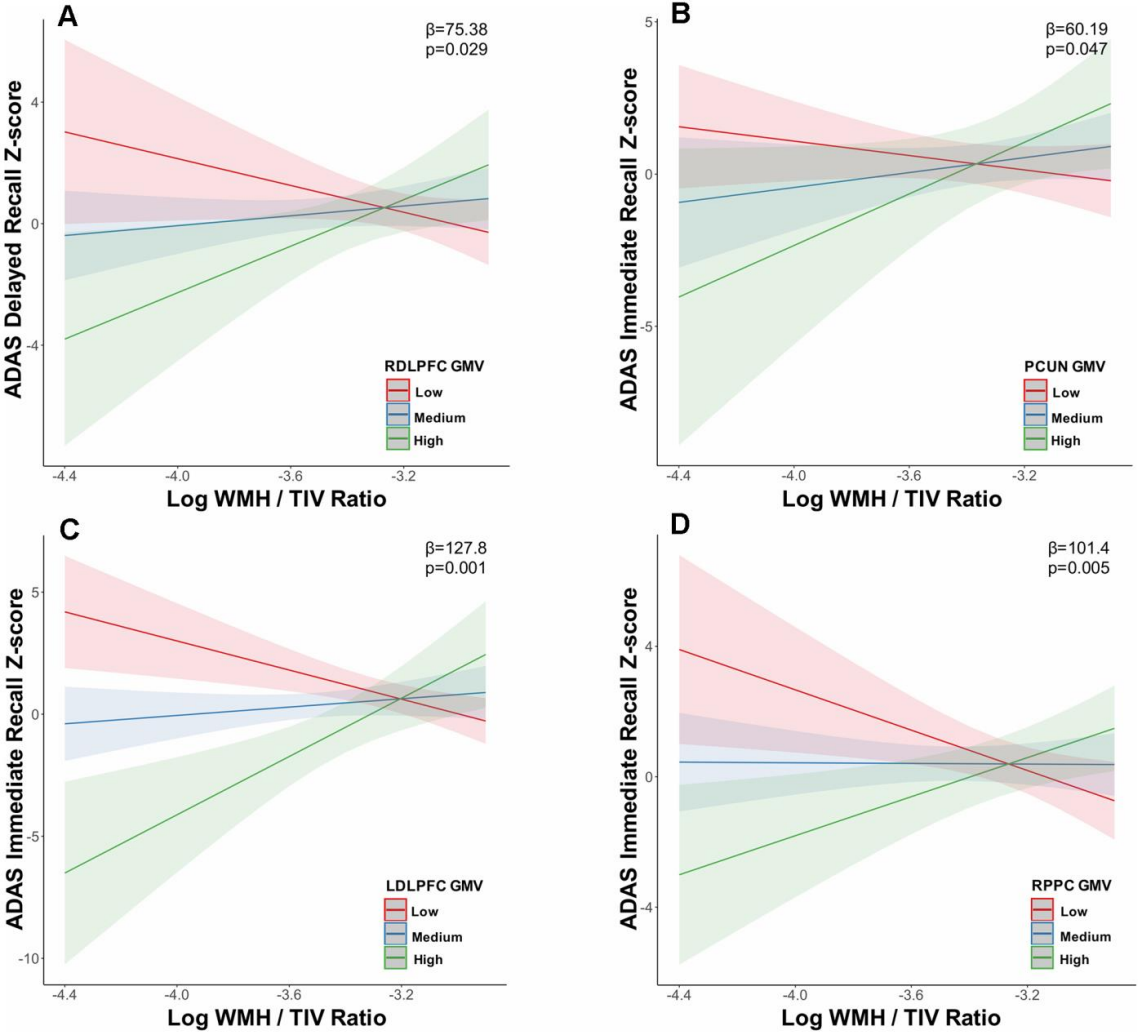
**Grey matter volume in the default mode and executive control networks moderates the relationship between white matter hyperintensity volume and memory at low white matter hyperintensity load.** In tercile 1, executive control network (**A**) RDLPFC, (**C**) LDLPFC, (**D**) RPPC and default mode network (**B**) PCUN grey matter volume moderated the relationship between memory (ADAS delayed recall, ADAS immediate recall) impairment and increasing WMH load. Abbreviations: ADAS, Alzheimer’s disease assessment scale; WMH, white matter hyperintensity; TIV, total intracranial volume; LDLPFC, left dorsolateral prefrontal cortex; RDLPFC, right dorsolateral prefrontal cortex; RPPC, right posterior parietal cortex; PCUN, precuneus; GMV, grey matter volume.

On the other hand, a moderation analyses revealed significant moderating effects of ROI GMV on the association between cognition and WMH load. Thus, ROI GMV interacted with WMH load to influence cognitive function. Specifically, at tercile 1, the interaction of increasing WMH load and RDLPFC GMV (β=75.38; uncorrected p<0.05; Figure 2A) related to worse performance on the ADAS delayed recall test. Similarly, the interaction of increasing WMH load and PCUN GMV (β=60.19; uncorrected p<0.05; Figure 2B) associated with worse performance on the ADAS immediate recall test. Higher WMH load and LDLFPC (β=127.80; p=0.001, FDR-corrected p<0.05; Figure 2C) and RPPC (β=101.40; p=0.0053, FDR-corrected p<0.05; Figure 2D) GMV related to worse performance on the ADAS immediate recall test. These results were controlled for age at diagnosis and sex.

No associations between WMH, GMV and memory/executive function were observed at tercile 2 in MCI.

**Figure 3:**
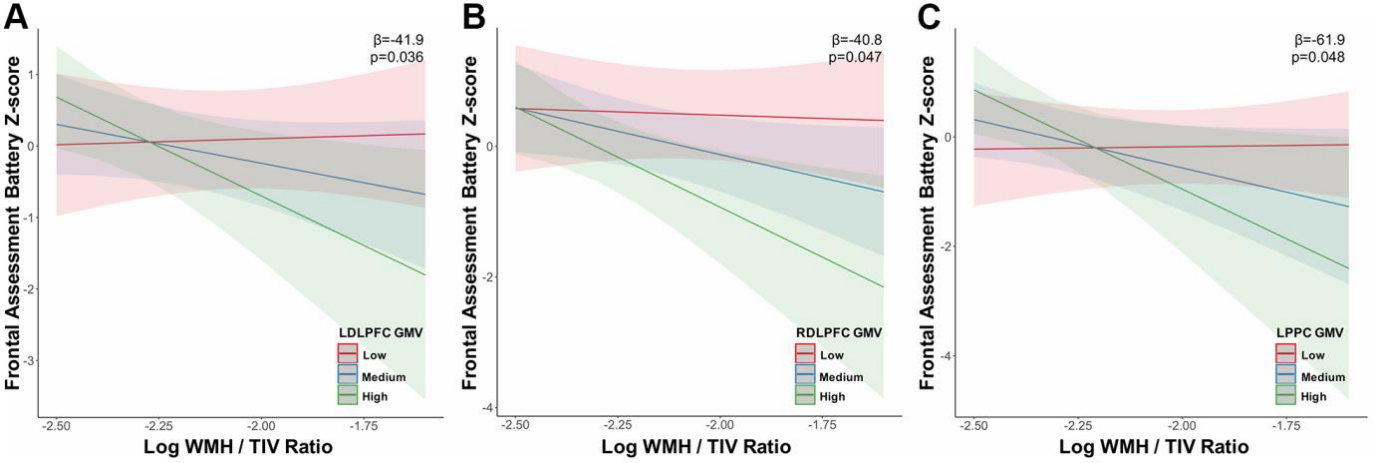
**Grey matter volume in the executive control network moderates the relationship between white matter hyperintensity volume and executive function at high white matter hyperintensity load.** The association between executive function (frontal assessment battery) decline and increasing WMH load in tercile 3 was moderated primarily by executive control network (**A**) LDLPFC, (**B**) RDLPFC and (**C**) LPPC grey matter volume. Abbreviations: WMH, white matter hyperintensity; TIV, total intracranial volume; LDLPFC, left dorsolateral prefrontal cortex; RDLPFC, right dorsolateral prefrontal cortex; LPPC, left posterior parietal cortex; GMV, grey matter volume.

**Figure 4:**
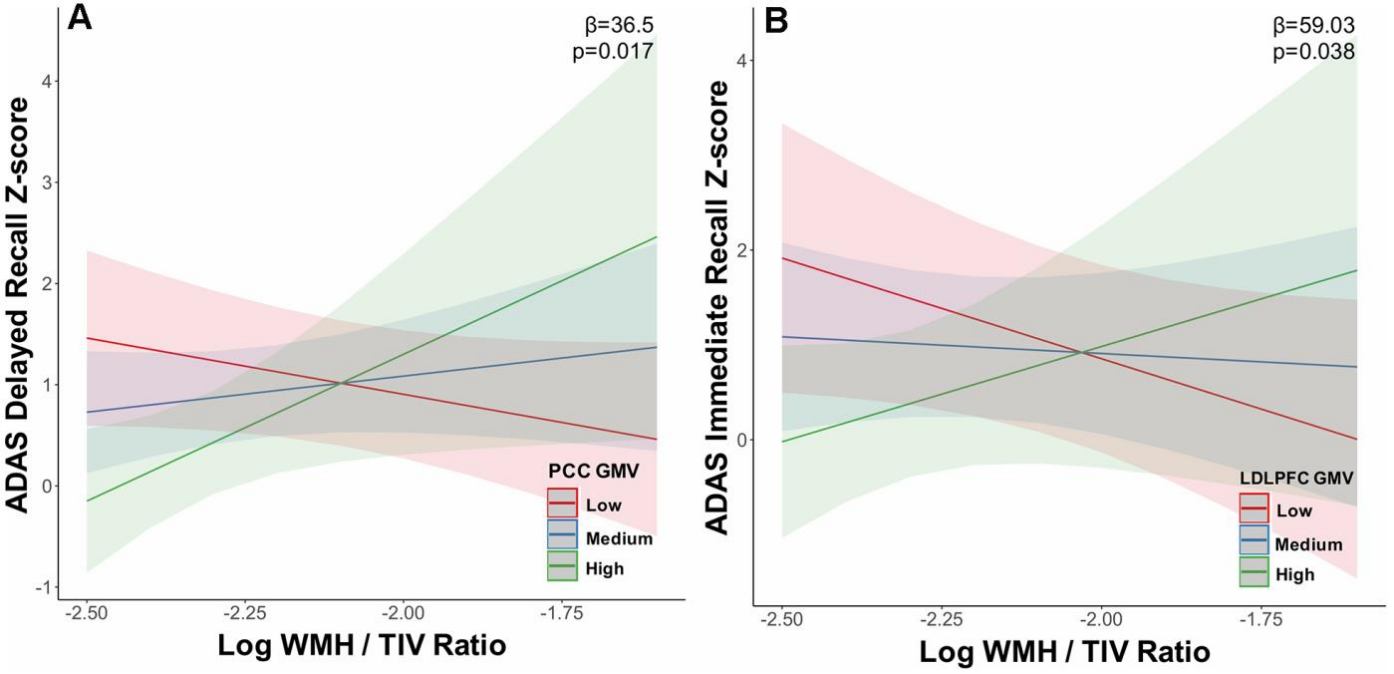
**Grey matter volume in the default mode and executive control networks moderates the relationship between white matter hyperintensity volume and memory at high white matter hyperintensity load.** In tercile 3, (**A**) default mode network PCC and (**B**) executive control network LDLPFC grey matter volume moderated the relationship between memory (ADAS delayed recall, ADAS immediate recall) impairment and increasing WMH load. Abbreviations: ADAS, Alzheimer’s disease assessment scale; WMH, white matter hyperintensity; TIV, total intracranial volume; LDLPFC, left dorsolateral prefrontal cortex; RDLPFC, PCC, posterior cingulate cortex; GMV, grey matter volume.

At tercile 3 i.e. the highest load of WMH, increasing WMH load interaction with ECN GMV related to lower FAB scores i.e. the LDLPFC (β=-41.9; uncorrected p<0.05; Figure 3A), RDLPFC (β=-40.8; uncorrected p<0.05) and LPPC (β=-61.90; uncorrected p<0.05; Figure 3B). Additionally, increasing WMH load interaction with PCC GMV (β=36.50; uncorrected p<0.05; Figure 4A) related to worse performance on the ADAS delayed recall and increasing WMH load interaction with LDLPFC GMV (β=59.03; uncorrected p<0.05; Figure 4B) related to worse performance on the ADAS immediate recall test. These results were controlled for age at diagnosis and sex.

## DISCUSSION

We demonstrate differential relationships between WMH load and voxel-wise and regional GM atrophy in the DMN and ECN with high WMH as hypothesized being negatively associated with GMV, however we additionally demonstrate low WMH to be positively associated with GMV. Such a differential relationship was only observed in the MCI stage with cognitively normal individuals showing consistently reduced GMV with all loads of WMH. Specifically, in MCI, low WMH load related to higher GMV in the ECN and lower voxel-wise frontoparietal GMV, however at higher WMH load, only a negative relationship was observed. At high WMH load, frontoparietal voxel-wise GMV and DMN and ECN ROI GMV demonstrated a negative relationship with increasing WMH burden. We also demonstrate GMV to be a key moderator in the relationship between WMH and cognition in MCI. Specifically, higher WMH was related to worse memory and executive function moderated by GMV in the DMN and ECN, while at low WMH load, only memory performance was affected. Our results highlight variable relationships between WMH and GMV, dependent on the amount of cerebral WMH burden and their subsequent association with cognition in participants with MCI, indicating that such associations cannot be assumed to be linear in nature. This variable relationship may have important implications in the clinical management of MCI patients with varying loads of WMH.

Cerebral WMH has been associated with several mechanisms including small vessel cerebrovascular disease, GM atrophy and neuroinflammation [13, 20, 25, 51, 52]. It has been widely assumed that irrespective of the underlying mechanism, increasing WMH load will result in progressive GMV loss. Our study demonstrates that this assumption is dependent on the baseline WMH load. While we demonstrate GMV decline in both the DMN and ECN ROI with high WMH volume, at low levels of WMH, this relationship is reversed and stage-dependent such that increasing WMH is associated with higher GMV, primarily in the ECN at the MCI stage. We additionally show, that at an intermediate load of WMH, the relationship between WMH load and GMV is stable and a negative relationship between increasing WMH load and ROI GMV is only observed when a certain threshold of WMH is reached. Our results are thus one of the first to shed light on a differential relationship between increasing WMH load and GMV, especially in the ECN, a network affected by the presence of cerebrovascular disease. In support of such findings, a few prior studies have illustrated increased cortical thickness related to the presence of WMH in older subjects without dementia [20, 53]. Increased cortical thickness has also been found in ageing studies likely indicative of local plasticity [54–56]. In patients with early stage cerebrovascular disease, inflammatory responses related to blood-brain barrier disruption may lead to the build-up of WMH accompanied by an increase in brain structural measures [52, 57]. Prior studies have also attempted to characterise compensatory task-based and resting-state functional MRI alterations associated with neural and cognitive aging. Compensatory ageing-related increases in activation in the DMN and parietal lobes are thought to reflect remodelling and neuroplasticity related processes to help support cognitive performance [58–60]. Moreover, the presence of WMH appears to elicit a neuroplastic response by stimulating grey matter adaptations and functional connectivity increases [20, 23]. An increase in number of synapses comprising synaptogenesis and increase in regional vasculature to compensate for ischaemia might be two possible mechanisms underlying increases in grey matter [23, 61]. Such changes would help maintain function in the presence of deleterious cerebrovascular disease [62]. Such processes may underlie the relative sparing of GMV at low and intermediate WMH loads in our study in early stages of disease. However, the functional implications of these associations still remain under conjecture and future longitudinal studies are needed to understand the trajectory of structural changes from the at-risk preclinical stage to established disease stage.

The finding of widespread negative association between WMH load and GMV at both the voxel-wise and ROI level at high WMH load evidenced in our study is supported by numerous prior studies [11, 13–16, 18–21, 25]. However, the mechanisms underlying this relationship need to be examined further. One possible mechanism includes anatomical disruptions due to the presence of subcortical WMH lesions subsequently leading to structural alterations of the cortex because of anterograde degeneration [63] as well as damage to specific white matter tracts connecting these regions [24, 64]. High cerebrovascular disease burden has also been shown to be related to have repercussions on brain structure and cognition through increase in amyloid-beta deposition or reduced amyloid-beta clearance [65, 66]. Additionally, changes in cortical structures themselves can lead to axonal loss and demyelination due to Wallerian degeneration [67]. Furthermore, the presence of WMH likely reflects microvascular damage, hypoperfusion and ischaemia within the cortex which may also underlie reduced GMV in overlapping regions and those connecting tracts affected by WMH [68, 69]. Notably, in the present study, the relationships between WMH and GMV remain unchanged after controlling for both hypertension status and systolic blood pressure, thus suggesting the involvement of other independent WMH-related factors. Thus, in line with and in addition to previous findings, our results lend evidence to differential load-dependent relationships between WMH and brain structure. Importantly, studies examining early stages of AD must take into account the presence and influence of co-morbid vascular risk factors as well as their potential interaction with AD-related brain pathology in order to determine their effects on disease progression.

The influence of WMH on cognition is predominantly thought to result in poor outcomes with prior studies showing reduction in executive function, memory and global cognition including perceptual speed [13, 26]. However, in our study there were no pair-wise associations between WMH and cognition, nor between GMV and cognition. Instead, the negative association between WMH and cognition was significant only in the presence of ECN and DMN GMV. Specifically, at tercile 1, memory decline was associated with increasing WMH moderated by GMV in the DMN and ECN. At tercile 3, more widespread associations were observed. Memory function and executive function were negatively associated with increasing WMH moderated by GMV in the DMN and ECN. Our results thus support the notion that increasing WMH burden is related to more widespread cognitive decline, with this relationship being strengthened by GMV loss in frontoparietal regions. These findings are in line with prior studies showing cortical atrophy mediating the relationship between WMH and cognition including memory [11, 20, 21, 29]. Since GMV moderated the relationship between WMH and cognition at both low and high WMH loads, it is likely that GMV is an important moderator regardless of WMH load. Additionally, increasing load of WMH, specifically periventricular WMH may result in damage to cholinergic neurotransmitter systems, and result in cognitive decline [70]. Thus, further studies assessing the effect of varying WMH load on cognition and the role of regional GMV on this association at various stages of disease are required.

The clinical relevance of our study may be that specifically in patients with MCI, presence of low burden of WMH may be indicative of an early stage of cortical dysfunction without neurodegeneration, wherein there is no grey matter loss, but instead there is compensatory grey matter increase [20, 53]. Detection of this stage (MCI with low burden of WMH) may provide a window of opportunity to institute interventions to retard the neurodegenerative process. However, when WMH load crosses a certain threshold, irreversible GMV loss begins and disease modifying interventions to slow GMV loss may be less beneficial.

### Limitations and future directions

Our study has several limitations. Since our analyses are based on cross-sectional data, our findings need to be further validated using a longitudinal dataset. Some of our findings did not pass multiple comparisons correction due to our moderate sample size though we used normalised data and data-driven methods to classify our levels of WMH load. In addition, although our MCI group was comprised of largely the amnestic sub-type, the presence of non-amnestic MCI subjects might confound the relationship between WMH, GMV and cognition. Notably, our cohort is representative of urban populations in Asia and worldwide. The generalizability of our findings to older populations and patients with lower education attainment will need to be studied further in future studies. Patients in our cohort were largely of Chinese ethnicity and future studies should focus on inclusion of more ethnicities to assess the impact of ethnicity on the relationship between WMH load, GMV and cognition. We also do not have a sufficient sample size to study this effect on patients with AD dementia. Moreover, the amyloid and tau status of our subjects was not known, thus we were unable to assess the influence of these AD biomarkers on the relationship between WMH and brain structure. Thus, future work will need to involve understanding the interaction between AD risk factors, WMH and GMV as well as the effect of white matter disruption on cognition across the AD spectrum.

## CONCLUSIONS

In summary we demonstrate differential effects of WMH burden on grey matter atrophy in the DMN and ECN in a load-dependent manner. Our results further shed light on the non-unidirectional relationship between WMH load, GMV and cognitive performance. Detection of MCI with low WMH, may enable targeted therapeutic interventions to delay neurodegenerative changes in ECN and DMN regions.
